# How to Select

**Published:** 1879-03

**Authors:** 


### HOW TO SELECT.

In the following substitute the word dentist for the word doctor, and the application will be as good in the one case as in the other.—[Ed. Register.

A contributer sends the following note on the proper rules for choosing a doctor. Not new and original, they are worth pondering by every doctor and patient:

Avoid a mean man, for you may be sure he will be a mean doctor, just as certain as he would make a mean husband,

Avoid a dishonest man; he will not be honest with you as your physician.

Shun the doctor that you can buy to help you out of a scrape—a good doctor can not be bought.

Avoid the untidy, coarse, blundering fellow, for the man who is clumsy in hitching his horse you may be sure is not handy at midwifery or surgery.

Avoid the doctor who flatters you and humors your appetites.

Avoid the empty blow-horn who blows of his numerous cases and tells you of seeing forty or fifty patients a day, while he spends two hours to convince you of the fact. Put him down as a fool.

To be a good doctor, one must first be a man in the true sense of the word.

He should be a moral man, honest in his dealings.

He must have good sense or he can not be a good doctor.

He should be strictly temperate. No one should trust his life in the hands of an intemperate doctor.

It is a good sign if he tells you how to keep well.

It is a good sign if the members of his own family respect him.—*Detroit Lancet.*

